# A case of relapsed pan-colonic ulcerative colitis accompanied with gastroduodenal lesions immediately after COVID-19

**DOI:** 10.1007/s12328-025-02107-0

**Published:** 2025-03-01

**Authors:** Katsuya Endo, Tomonori Satoh, Yuki Yoshino, Shiho Kondo, Yoko Kawakami, Daisuke Fukushi, Atsuko Takasu, Takayuki Kogure, Morihisa Hirota, Kennichi Satoh

**Affiliations:** Division of Gastroenterology, Tohoku Medical and Pharmaceutical University School of Medicine, 1-15-1 Fukumuro, Miyagino-ku, Sendai, Miyagi 983-8536 Japan

**Keywords:** Ulcerative colitis, UC-associated gastroduodenal lesion, COVID-19

## Abstract

Some patients with ulcerative colitis (UC) suffer from complicated UC-associated upper gastrointestinal lesions. However, the trigger of these lesions has not been clarified. Herein, we present a 28-year-old man with relapsed pan-colonic UC accompanied by gastroduodenal lesions immediately after contracting coronavirus disease 2019 (COVID-19). In this patient, UC relapsed approximately 7 days after the COVID-19 onset, despite being in remission for 3 years. The patient also developed symptoms such as epigastric pain and nausea on day 19 of COVID-19. The endoscopic and pathologic findings of the stomach and duodenum closely resembled colorectal lesions of UC; accordingly, we diagnosed the patient with UC-associated gastroduodenal lesions. Corticosteroids were significantly effective in the colorectal and upper gastrointestinal lesions, leading to remission. This report is the first to describe UC-associated upper GI lesions that developed right after COVID-19 infection. Therefore, COVID-19 can be a possible trigger of UC-associated upper gastrointestinal lesion. Further studies are needed to understand the relationship between the onset of UC or UC-associated upper GI lesions and COVID-19.

## Introduction

Ulcerative colitis (UC) is a chronic inflammatory disease of the large intestine with unknown etiology. However, some reports have described UC cases accompanied by upper gastrointestinal (GI) tract inflammation. UC-associated upper GI lesions are characterized by diffuse inflammatory lesions in the stomach, duodenum, and/or intestine that resemble colonic lesions of UC in terms of endoscopic and pathological findings [1]. However, the trigger of UC-associated upper GI lesions remains unclear.

The novel coronavirus disease 2019 (COVID-19) induced by severe acute respiratory syndrome coronavirus-2 (SARS-CoV-2) has spread worldwide since the end of 2019. However, only a few reports have described cases of UC triggered by COVID-19 [2–4].

Herein, we report for the first time a rare case of relapsed pan-colonic UC accompanied by UC-associated gastroduodenal lesions immediately after contracting COVID-19.

## Case report

A 28-year-old man presented to our hospital complaining of fever, sore throat, and cough. He was diagnosed with COVID-19.

**Fig. 1:**
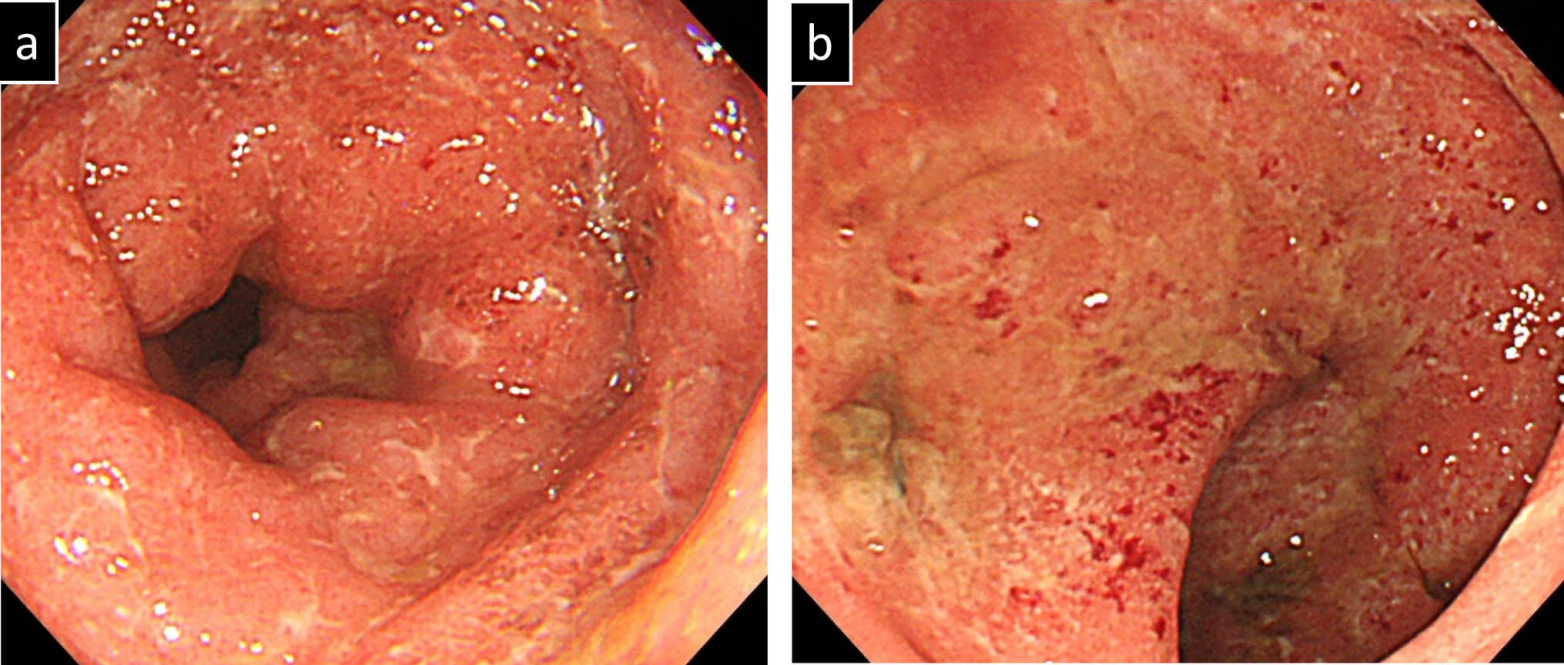
Features observed in colonoscopy at the time of initial diagnosis of UC. (**a**) Sigmoid colon. (**b**) Rectum. Continuous extension of erythema, edema, and erosion with adherent pus from the rectum to the oral side was observed. These findings were consistent with moderate ulcerative colitis

The patient had a history of pancolitis-type ulcerative colitis diagnosed three years earlier at another hospital. Colonoscopic findings at the time of initial diagnosis are shown in Fig. 1. According to medical records from that institution, the patient had an allergy to 5-aminosalicylic acid (5-ASA) and experienced spontaneous remission upon its discontinuation. Subsequently, the patient independently discontinued follow-up visits, and no further detailed evaluations, including endoscopy, were performed. However, he remained asymptomatic for UC for 3 years.

**Fig. 2:**
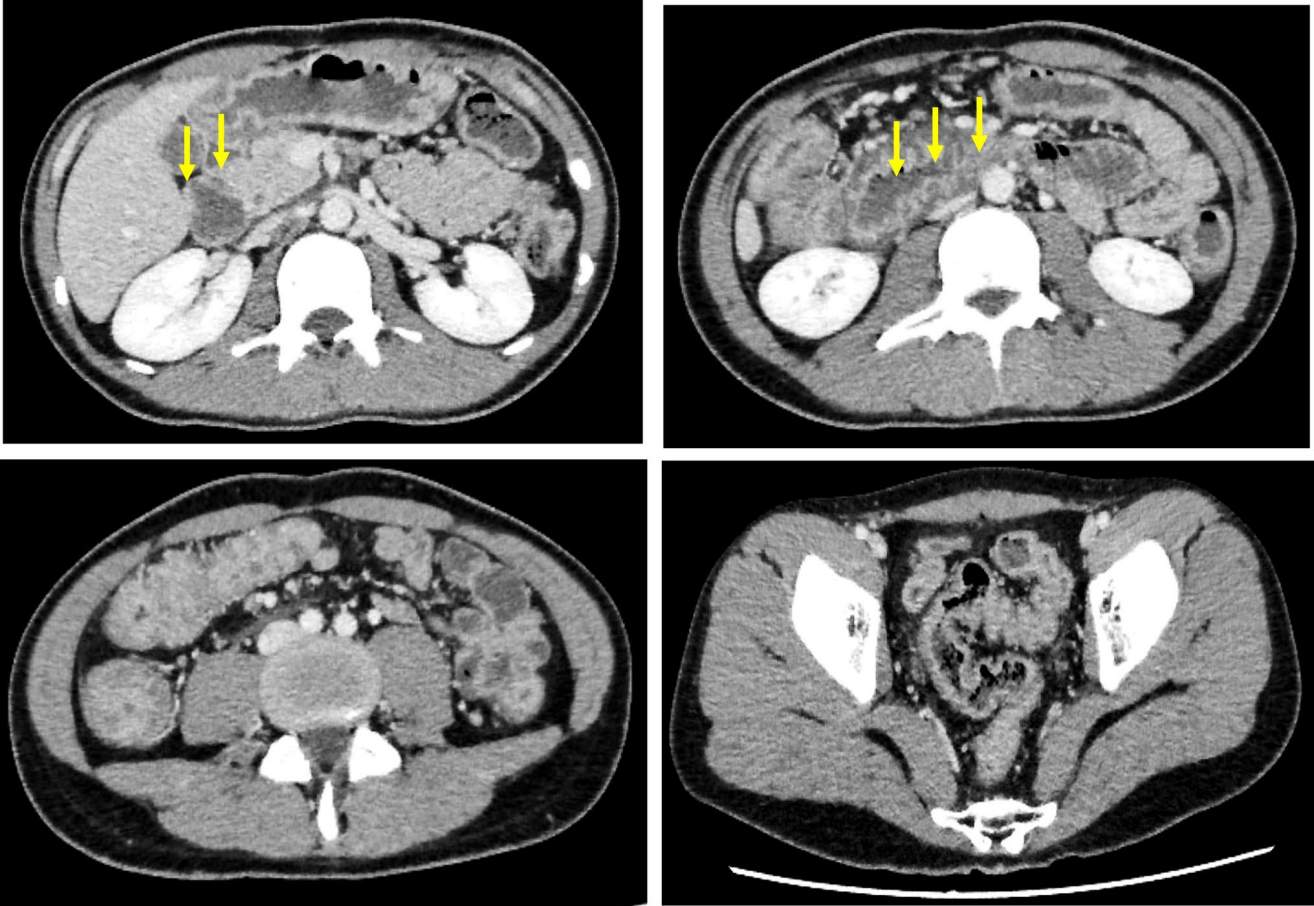
Contrast-enhanced computed tomography of the abdomen.The entire colon and the duodenum showed enhanced marked wall thickening (arrow)

**Fig. 3:**
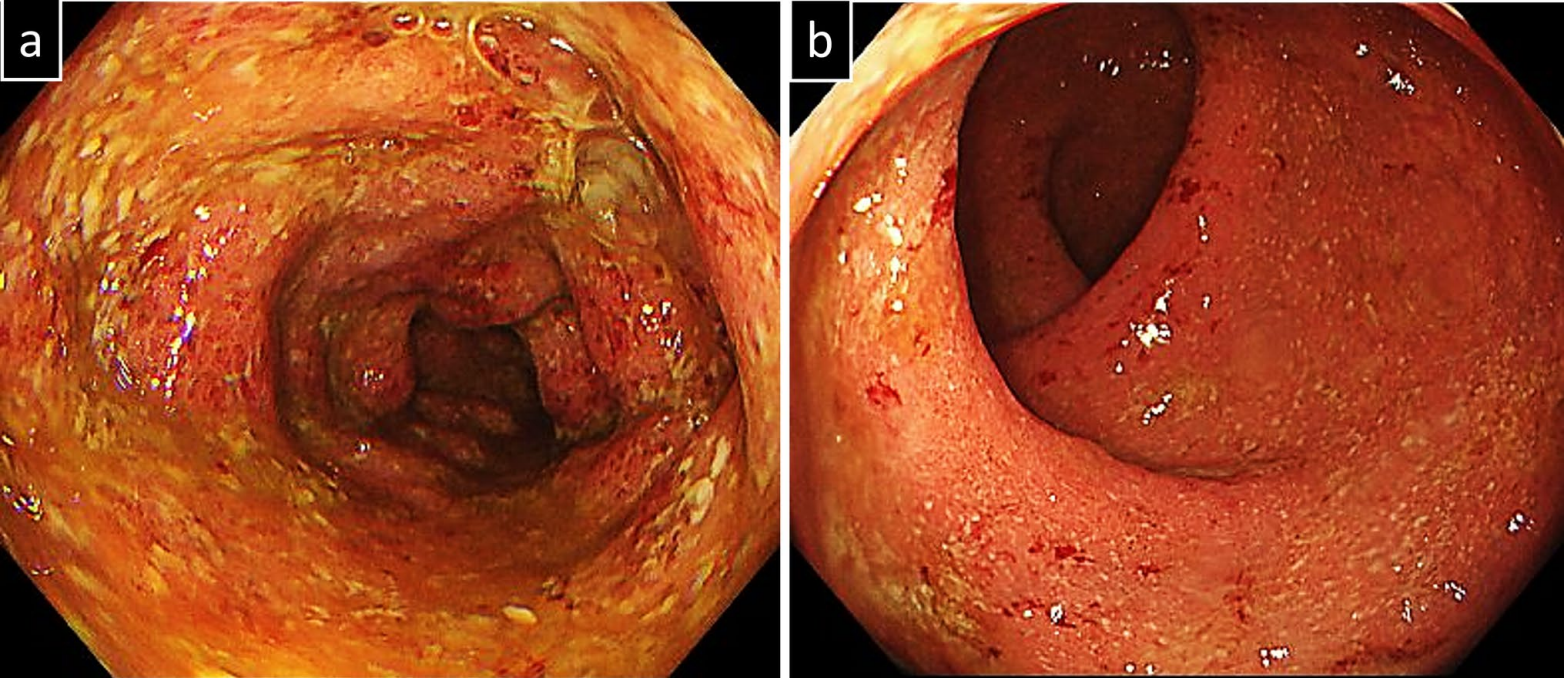
Features observed in colonoscopy on day 21 of COVID-19 onset. **a** Descending colon. **b** Rectum. Colonoscopy revealed edematous mucosa, erythema, obliterated vascular pattern, luminal mild bleeding, and erosions, which all indicate moderately active UC. These appearances were circumferential and continuous from the rectum to the transverse colon

**Fig. 4:**
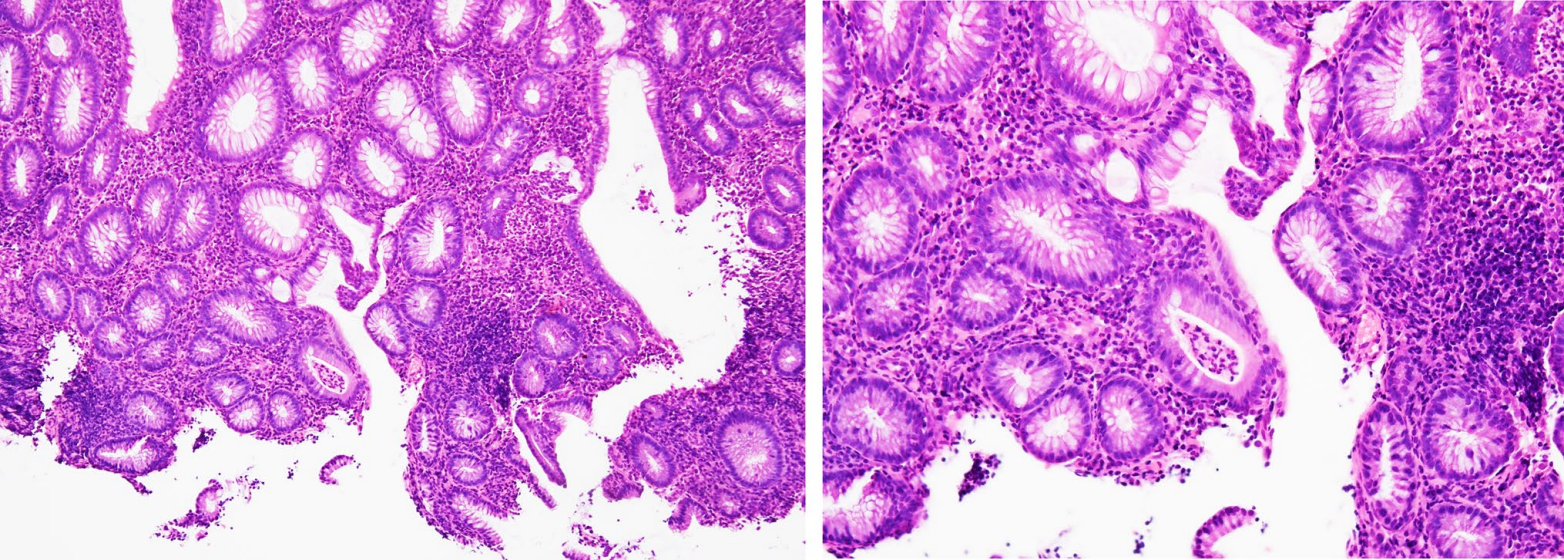
Pathological findings of the mucosal biopsies from the rectum. The biopsy specimens extracted from the rectum showed inflammatory findings including neutrophilic infiltration, cryptitis, and crypt abscess

**Fig. 5:**
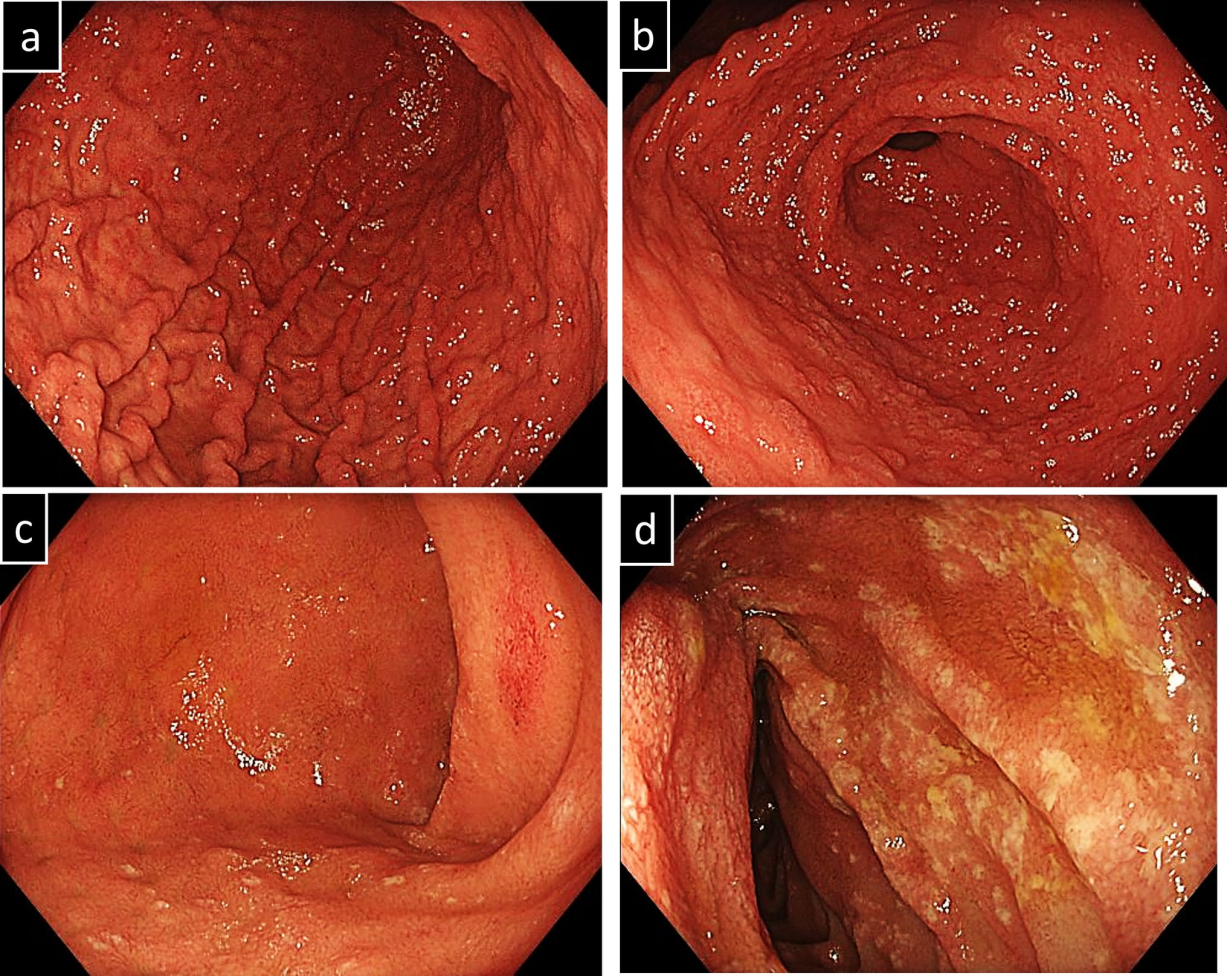
Features observed in esophagogastroduodenoscopy on day 22 of COVID-19 onset. Erythema, edema, and granular mucosa were detected in the stomach, mainly in the lower gastric body and antrum (**a**,**b**). Erythema, edema, erosion, and coarse mucosa with pus adhesion were noted in the duodenum (**c**,**d**)

**Fig. 6:**
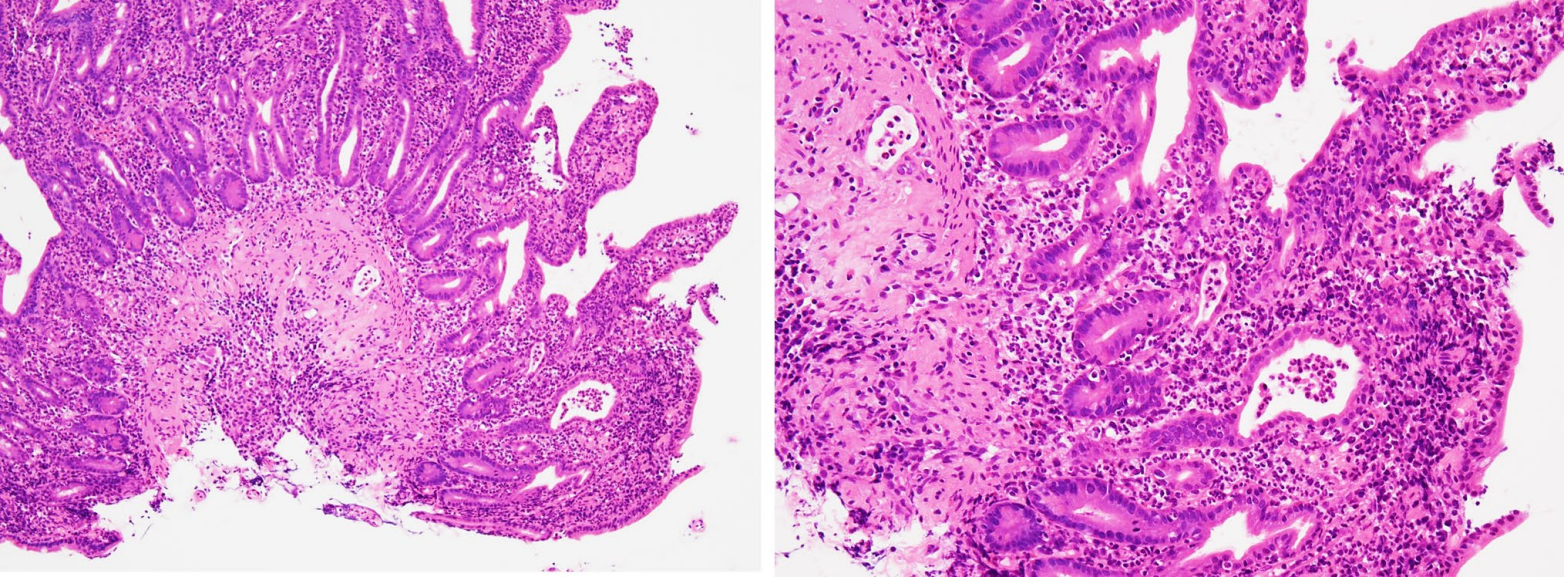
Pathological findings of the mucosal biopsies from the duodenum. The biopsy specimens from the duodenum showed inflammatory findings similar to those in the rectum, including neutrophilic infiltration, cryptitis, and crypt abscesses

Then, he was diagnosed with COVID-19, but his fever and upper respiratory tract symptoms improved on day 5 of infection by restful recuperation and symptomatic treatment. However, on day 7, he developed a fever of 38℃, abdominal pain, and bloody stool. On day 19, he also developed severe epigastric pain and nausea, prompting admission to our hospital. The laboratory findings indicated elevated white blood cell count, decreased hemoglobin level, increased platelet count, marked hypoalbuminemia, and elevated C-reactive protein level (Table 1). Contrast-enhanced computed tomography of the abdomen showed marked wall thickening of the entire colon and duodenum (Fig. 2). In contrast to the marked thickening observed in the duodenum and large intestine, the CT scan showed no wall thickening in the jejunum and ileum. On day 21, a colonoscopy was performed, inserting an endoscope into the transverse colon for observation. The colonoscopy revealed edematous mucosa, erythema, obliterated vascular pattern, luminal mild bleeding, and erosions, indicating moderately active UC (Fig. 3). Pathological findings of biopsy specimens from the rectum showed neutrophilic infiltration, cryptitis, and crypt abscess, which were also consistent with UC (Fig. 4). To exclude bacterial enteritis, we performed a stool culture, which did not reveal any significant bacterial growth. Additionally, both the *Clostridioides difficile* antigen and toxin tests were negative, ruling out *Clostridioides difficile* infection. According to these findings, we diagnosed the patient with relapsed pan-colonic UC with moderate severity. On day 22, the patient underwent esophagogastroduodenoscopy (EGD) because the patient complained of severe epigastric pain and nausea. Erythema, edema, and granular mucosa were noted in the stomach, mainly in the lower gastric body and antrum (Fig. 5 a, b). Additionally, the duodenum exhibited erythema, edema, erosion, and coarse mucosa with pus adhesion (Fig. 5 c,d). Biopsy specimens from the duodenum showed histopathologic findings suggestive of colonic lesions of UC, including inflammatory cell infiltration, glandular duct atrophy, disorganized arrangement, and crypt abscess (Fig. 6). After ruling out intestinal infection such as cytomegalovirus (CMV) infection and vasculitis, we diagnosed him with UC-associated gastroduodenal lesion according to the upper GI findings.

**Table 1:** Laboratory data on admission

WBC	11,200	/µL	BUN	10	mg/dL
RBC	440	× 10^4^/µL	Cr	0.59	mg/dL
Hb	13	g/dL	Na	134	mEq/L
Ht	38.1	%	K	3.1	mEq/L
MCV	86.5	fL	Cl	67	mEq/L
MCH	29.6	pg	Ca	8.3	mg/dL
MCHC	34.2	%	TP	5.2	g/dL
Plt	63.8	× 10^4^/µL	Alb	2.1	g/dL
AST	10	IU/L	T-Chol	94	mg/dL
ALT	7	IU/L	TG	89	mg/dL
LDH	145	IU/L	FBS	100	mg/dL
T-Bil	0.28	mg/dL	CRP	6.53	mg/dL
ALP	55	U/L			
gGTP	19	IU/L			

*WBC* white bold cells, *RBC* red blood cells, *Hb* hemoglobin, *Ht* hematocrit, *MCV* mean corpuscular volume, *MCH* mean corpuscular hemoglobin, *MCHC* mean corpuscular hemoglobin concentration, *Plt* platelets, *AST* aspartate aminotransferase, *ALT* alanine aminotransferase, *LDH* lactate dehydrogenase, *T-Bil* total bilirubin, *ALP* alkaline phosphatase, *gGTP* gamma-glutamyltranspeptidase, *S-AMY* serum amylase, *BUN* blood urea nitrogen, *Cr* creatinine, *Na* sodium, *K* potassium, *Cl* chloride, *Ca* calcium, *TP* total protein, *Alb* albumin, *T-Chol* total cholesterol, *TG* triglycerides, *FBS* fasting blood sugar, *CRP* C-reactive protein

**Fig. 7:**
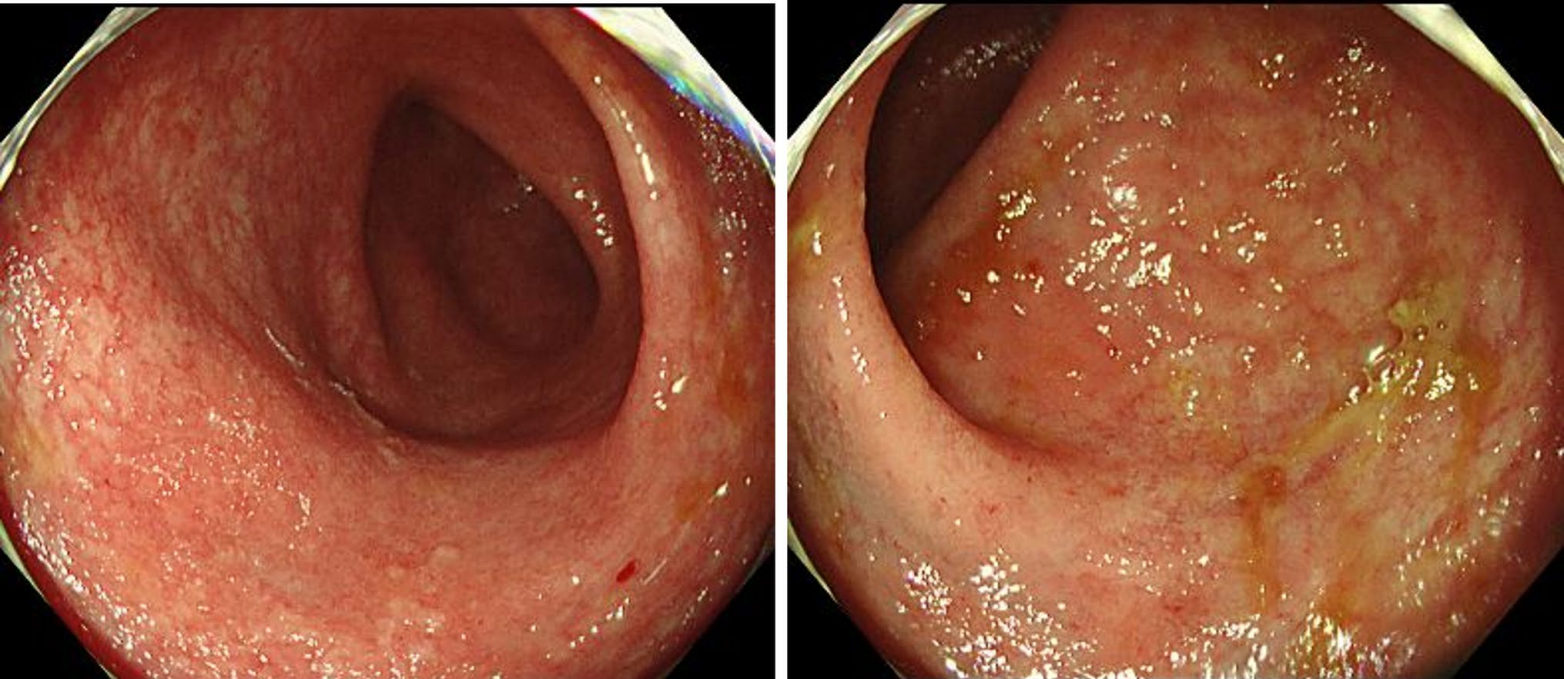
Features observed in colonoscopy 2 weeks after PSL administration. Sigmoid colonoscopy performed 2 weeks after PSL administration showed significantly improved mucosal findings.

**Fig. 8:**
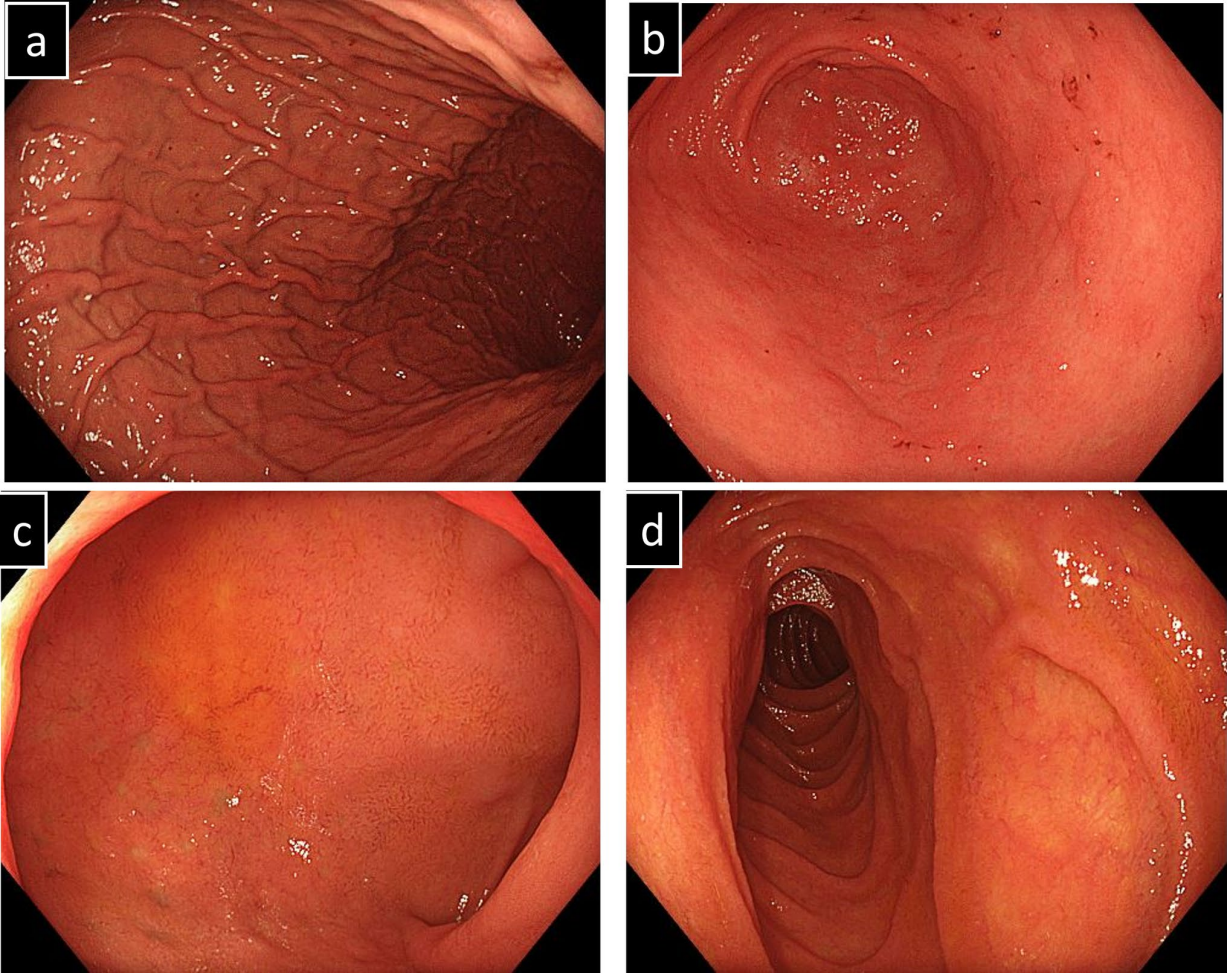
Features observed in esophagogastroduodenoscopy 2 weeks after PSL administration. Esophagogastroduodenoscopy performed 2 weeks after PSL administration showed significantly improved mucosal findings

**Fig. 9:**
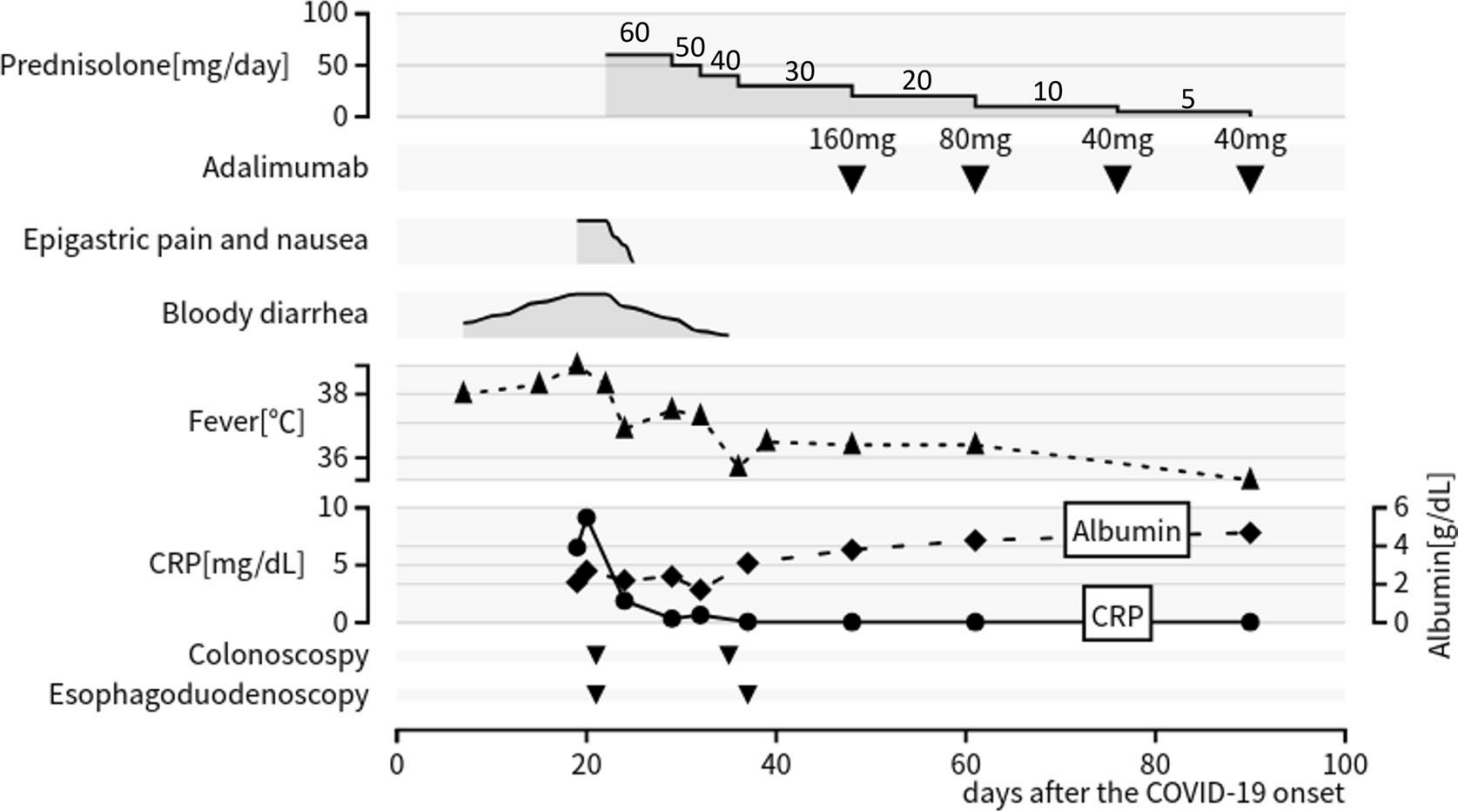
Clinical course after the COVID-19 onset

We initiated remission induction therapy by intravenously injecting prednisolone (PSL, 60 mg/day). The upper and lower GI tract symptoms improved rapidly after PSL administration, and clinical remission was achieved after 2 weeks. SCS and EGD performed 2 weeks after PSL administration showed significantly improved mucosal findings (Fig. 7, 8). On day 18 of PSL administration, he was discharged with outpatient management. PSL was systematically tapered, and adalimumab was started for remission maintenance because of 5-aminosalicylic acid allergy. With the introduction of adalimumab, he became steroid-free, and his UC and duodenal lesions have not relapsed for more than 1 year on either therapy. The clinical course after the COVID-19 onset is presented in Fig. 9.

## Discussion

The disease extent of UC is usually confined to the colon and the rectum. However, the involvement of the upper GI tract has been recently reported [1, 5–11]. UC-associated upper GI lesions are characterized by diffuse inflammatory lesions in the stomach, duodenum, and/or intestine that resemble colonic lesions of UC on the basis of both endoscopic and pathological findings. In the present case, we suspected upper GI lesions because of the presence of epigastric pain and nausea, which are not usually seen as UC symptoms. The endoscopic and pathologic findings of the stomach and duodenum in our case are closely similar to the colorectal lesions of UC and consistent with UC-associated upper GI lesions. In the diagnosis of UC-associated upper GI lesions, other diseases, such as infectious disease and systemic vasculitis, must be excluded [8, 12, 13]. In the present case, CMV infection was ruled out according to the negative histopathology specimen and negative blood CMV antigenemia. Systemic IgA vasculitis was also ruled out because of the absence of systemic symptoms such as purpura, arthralgia, and renal involvement. Small bowel inflammation caused by COVID-19 is also an important consideration in the differential diagnosis. The CT scan of this patient showed no wall thickening in the jejunum and ileum, in contrast to the marked thickening observed in the duodenum and large intestine. Therefore, endoscopic examination of the small intestine distal to the duodenum was not performed. The colorectal lesions exhibited typical relapsing UC features, while the gastroduodenal lesions demonstrated UC-like endoscopic and histopathologic findings. Given these observations, we concluded that enteritis related to COVID-19 was deemed unlikely, and the diagnosis was consistent with upper GI involvement of UC.

While the treatment strategy for UC-associated upper GI lesions remains poorly established, these lesions are empirically known to improve by treatment for UC. Similar to treatment for colitis in UC, 5-aminosalicylic acid, corticosteroid, and anti-TNF alfa antibody can be used for UC-associated upper GI lesions or enteritis [7, 12–17]. In the present case, the good response to steroid treatment strongly supports that these lesions are UC-associated upper GI lesions. In this case, adalimumab was initiated following corticosteroid treatment. There were two primary reasons for selecting anti-TNF-α therapy. First, the patient had a history of 5-ASA allergy, which limited the available options for effective maintenance therapy. In the absence of 5-ASA treatment, the patient was considered at high risk for relapse. Therefore, we opted for an anti-TNF-α agent to ensure sustained remission. Second, anti-TNF-α antibodies are an appropriate treatment for active UC complicated by COVID-19 infection and are not considered a significant risk factor requiring special precautions. According to recent evidence, Nakase et al. analyzed Japanese IBD patients with COVID-19 and reported that immunosuppressive therapies other than corticosteroids (including thiopurines and anti-TNF-α agents) are associated with a relatively low risk of severe COVID-19 infection [18]. Furthermore, the British Society of Gastroenterology (BSG) guidelines recommend the combination of corticosteroids and infliximab for patients with severe UC during COVID-19 infection [19]. Based on these findings, we determined that anti-TNF-α antibody therapy would be appropriate for managing COVID-19-induced UC relapse. In the present case, the introduction of adalimumab proved to be an effective therapeutic strategy, as it did not exacerbate COVID-19 infection and successfully induced and maintained remission of UC with upper GI involvement.

The triggers of upper GI inflammation in UC are still unclear. Most of the reported cases of UC-associated GI lesions were accompanied by pan-colonic UC, indicating that pan-colonic inflammation is strongly associated with upper GI inflammation [5, 6, 10]. A lower dose of prednisolone has been also reported as a possible risk factor for developing UC-associated upper GI lesions [5]. In addition, total or subtotal colectomy is one of the important triggers for the development of UC-associated upper GI lesions and small bowel lesions [8, 9, 11, 20]. Kohyama et al. reported that 42 (0.8%) out of 5284 UC cases had been diagnosed with UC-related postoperative enteritis [9]. However, the other triggers of UC-associated upper GI lesions remain largely unknown. The total colitis observed in our patient is similar to that seen in previously reported cases. Of note, in our case, the relapse of UC and the appearance of upper GI lesions occurred almost concurrently, immediately after COVID-19. A recent European cohort study has reported that COVID-19 has a slight impact on the course of inflammatory bowel disease (IBD) [21]. However, a multicenter study from Japan has described that COVID-19 changed the course of IBD in approximately 10% of patients [22]. Furthermore, the onset of UC triggered by COVID-19 has been reported in some patients [2–4]. Although evidence is still limited, these reports suggest that COVID-19 amplifies UC or causes UC by revealing masked inflammation. In the present case, UC relapsed approximately 7 days after the COVID-19 onset, despite being in remission for 3 years. The patient also developed UC-associated gastroduodenal lesions almost at the same time as the UC recurrence. Thus, the activation of the systemic immune system by COVID-19 may have caused the recurrence of UC and the development of UC-related upper GI lesions.

In conclusion, this report describes for the first time a rare case of relapsed pan-colonic UC accompanied by UC-associated GI lesions immediately after contracting COVID-19. COVID-19 can be a possible trigger of UC-associated upper GI lesion. In patients with UC infected with COVID-19, we should be aware of the development of upper GI lesions and the relapse or worsening of UC. Further studies are needed to understand the relationship between UC or UC-associated upper GI lesion onset and COVID-19.
